# Lipschitz-based robustness estimation for hyperdimensional learning

**DOI:** 10.3389/frai.2025.1637105

**Published:** 2025-09-17

**Authors:** Calvin Yeung, Hamza Errahmouni Barkam, Zhuowen Zou, Sanggeon Yun, Nathaniel D. Bastian, Mohsen Imani

**Affiliations:** ^1^Department of Computer Science, University of California, Irvine, Irvine, CA, United States; ^2^Department of Electrical Engineering & Computer Science, United States Military Academy, West Point, NY, United States

**Keywords:** hyperdimensional computing, vector symbolic architectures, robustness, adversarial attacks, classification

## Abstract

With the adoption of machine learning models in various practical domains, there is a growing need for evaluating and increasing model robustness. Hyperdimensional computing (HDC) is a neurosymbolic computational paradigm that represents symbols as high dimensional vectors and symbolic operations as vector operations, seamlessly interfacing between neuro- and symbolic components of a model. However, there is a notable gap in HDC research regarding the robustness of HDC models to input perturbations. This study presents a novel theoretical framework tailored to evaluate the robustness of hyperdimensional classifiers against perturbations in the input space. In particular, our proposed measure of robustness gives a theoretical upper bound for the magnitude of noise a model can tolerate without changing its prediction for any given data point. We also propose a method to enhance the robustness of the model based on our proposed measure of robustness. Our approach introduces several methods to calculate model robustness as a function of the specific dataset and type of hyperdimensional encoding used. The results show that the average robustness of HDC models increases under the proposed optimization scheme while maintaining accuracy by varying the variance of the Gaussian distribution used to encode hypervectors. The practical effectiveness of our proposed measure of robustness is also demonstrated.

## 1 Introduction

With the adoption of machine learning models in various practical domains, there is a growing need for methods that evaluate and increase model robustness, as models may be susceptible to noise, whether in the model representation or in the model input, due to various reasons such as adversarial attacks or a noisy environment or hardware. For this reason, there has been a plethora of empirical and theoretical studies on the robustness of deep learning models (Cisse et al., 2017; Wong and Kolter, 2018a; Raghunathan et al., 2018; Cohen et al., 2019; Wong and Kolter, 2018b; Zhang et al., 2019).

**Figure 1:**
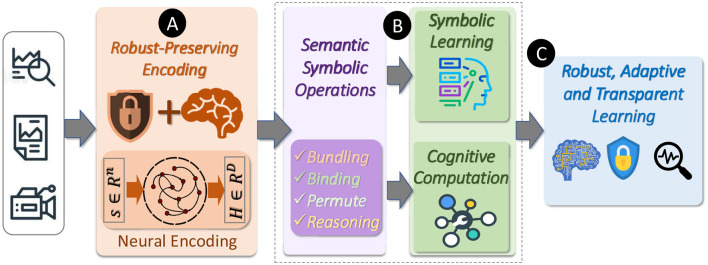
HDC encodes data and symbols as hypervectors **(A)** which can be manipulated using vector operations in a symbolic manner **(B)**, providing robustness and interpretability **(C)**.

However, deep learning methods remain difficult to interpret and have difficulty performing symbolic reasoning. Neurosymbolic methods address these gaps by integrating neural networks with a symbolic component. Hyperdimensional computing (HDC) has emerged as a promising neurosymbolic computational paradigm, capable of both machine learning and cognitive reasoning tasks. In HDC, symbols are represented as high-dimensional vectors called hypervectors. Symbolic operations thus correspond to vector operations such as bundling and binding. The vector representation of symbols in HDC provides a natural interface with deep networks. As summarized in Figure 1, HDC encodes data as hypervectors (Figure 1A) and manipulates them with symbolic operations such as bundling and binding (Figure 1B), yielding models that are robust and interpretable (Figure 1C).

HDC has been successfully applied to a variety of machine learning tasks, including classification and regression, and has shown to have performance comparable to neural networks on simple tasks while achieving higher noise tolerance to perturbations in model representations, higher transparency, lower power consumption, and intrinsic one- or few-shot learning capabilities (Kleyko et al., 2023; Kymn et al., 2024; Hernández-Cano et al., 2021; Poduval et al., 2022; Yeung et al., 2025). Despite the success of HDC in machine learning tasks, most work in applying HDC to machine learning has focused on model performance in terms of accuracy metrics. While there have been theoretical studies on the robustness of HDC models to noise in hyperdimensional space (Thomas et al., 2022), there is a lack of theoretical work on that in the input space.

This study aims to fill this gap by providing theoretically supported robustness measures in HDC classifiers. In particular, in this work, we focus on the robustness of a model to perturbations on the input space. To this end, we use a Lipschitz-based approach to derive robustness estimates for HDC classifiers. Our work provides a first-of-its-kind theoretical framework for evaluating model robustness to input perturbations in HDC and a novel method for learning encodings that are more robust. Our results demonstrate that our method can be used effectively to derive robustness estimates for HDC classifiers and that our method for learning more robust encodings can improve the robustness of these models. This study makes several key contributions: (1) We propose a first-of-its-kind method that characterizes theoretical per-point robustness for HDC classifiers based on Lipschitz continuity; (2) We introduce two methods for estimating the Lipschitz constant, with one that gives more liberal estimates and another that gives more conservative estimates; (3) We provide a general framework for learning a robust encoding based on the estimates of robustness we have derived.

## 2 Related work

In the deep neural network (DNN) literature, there is a body of work that explores the kind of robustness we are concerned with in this study, i.e., the robustness of classifiers to input perturbations from an adversarial perspective (Cisse et al., 2017; Wong and Kolter, 2018a; Raghunathan et al., 2018; Cohen et al., 2019; Wong and Kolter, 2018b; Zhang et al., 2019). In particular, multiple studies have explored the certified robustness of classifiers, which provides theoretical estimates for the robustness of DNNs to norm-bounded perturbations in the input. Cisse et al. (2017) give an upper bound for the generalization bound on the loss function that accounts for adversarial examples with perturbations up to a given magnitude in terms of the Lipschitz constant of the neural network and proposes Parseval regularization, which constrains the Lipschitz constant of each hidden layer to be less than one, to increase the robustness of the DNN.

In the HDC literature, there have been more studies investigating the robustness of HDC models to perturbations in some parts of the model representation (Thomas et al., 2022; Zhang et al., 2021; Rahimi et al., 2016; Matsui et al., 2023). Zhang et al. (2021) explore the robustness of HDC models to errors in associative memory by injecting errors into class hypervector representations and measuring the degradation of model accuracy. Thomas et al. (2022) give HDC a theoretical treatment and explore the robustness of HDC models to perturbations in hyperspace for decoding and learning tasks but do not provide a treatment for perturbations in the input space.

To the best of our knowledge, our work is the first of its kind to investigate the theoretical robustness of HDC classifiers to perturbations in the input space. At a high level, it is inspired by works in certified defenses in DNNs but is explicitly catered to HDC, using HDC theory in the analysis.

## 3 Background

HDC, also known as vector symbolic architecture, is a computing framework based on properties of high dimensional vectors. The fundamental unit in HDC is a high dimensional vector, also called a hypervector. A hypervector H lives in some hyperspace *H*, e.g., ℝ^*D*^ for *D* large, and, together with some operations in hyperspace, form an algebra over vectors. Generally, there are two types of hypervectors: (1) base hypervectors, which are generated stochastically, e.g., H~N(0,I); and (2) composite hypervectors, which are created by combining hypervectors using a variety of operations. These hypervectors can be compared via a similarity operation $\delta \left(H_{1},H_{2}\right)$. In this work, we are mainly concerned with the inner product as a similarity measure. Generally, basic hypervectors are generated to be mutually dissimilar; i.e., quasi-orthogonal.

The three main operations in HDC, namely, bundling, binding, and permutation, can be characterized by how it affects the similarity of hypervectors. Bundling, denoted as +, is typically implemented as element-wise addition. If $H=H_{1}+H_{2}$, then both $H_{1}$ and $H_{2}$ are similar to H. From a cognitive perspective, it can be interpreted as memorization. Binding, denoted as *, is typically implemented as element-wise multiplication. If $H=H_{1}*H_{2}$, then H is dissimilar to both $H_{1}$ and $H_{2}$. Binding also has the important property of similarity preservation in the sense that for some hypervector V, $\delta \left(V*H_{1},V*H_{2}\right)≃\delta \left(H_{1},H_{2}\right)$. From a cognitive perspective, it can be interpreted as association. Permutation, denoted as ρ, is typically implemented as a rotation of vector elements. Generally, δ(ρ(H),H)≃0. Permutation is usually used to encode order in sequences.

It is important to note that the description above of HDC is general; there are various specific realizations of HDC with the above properties. The HDC framework gives several benefits, including robustness to noise in hyperspace, transparency, and parallelization.

### 3.1 Hyperdimensional learning

In this subsection, we discuss learning in HDC, focusing on classification. To adapt HDC to the task of learning from some dataset D⊂U, where *U* is the input space, we must define an encoding ϕ:*U*→*H* that preserves some notion of similarity in the input space. Thus, given some input *x, y*∈*U*, ϕ(*x*), ϕ(*y*) are their corresponding hypervector, and ϕ(*x*) is similar to ϕ(*y*) if and only if *x* is similar to *y*.

Suppose our dataset D consists of *m* classes *C*_1_, *C*_2_, …, *C*_*m*_, where $C_{i}=\left\{x_{1}^{\left(i\right)},x_{2}^{\left(i\right)},...,x_{N_{i}}^{\left(i\right)}\right\}$ for 1 ≤ *i* ≤ *m*. Based on these classes, We can define a class hypervector ϕ(*C*_*i*_)^1^ for 1 ≤ *i* ≤ *m*. A simple way to form a class hypervector is to simply bundle all hypervectors corresponding to elements in the class; i.e., $\phi \left(C_{i}\right)=\underset{x\in C_{i}}{\sum }\phi \left(x\right)$ for 1 ≤ *i* ≤ *m*. There are various other ways of forming class hypervectors which have the general form of a weighted sum $\phi \left(C_{i}\right)=\overset{N_{i}}{\underset{k=1}{\sum }}\gamma_{k}^{\left(i\right)}\phi \left(x_{k}^{\left(i\right)}\right)$, where $\gamma_{k}^{\left(i\right)}\in \mathbb{R}$ for all 1 ≤ *k* ≤ *N*_*i*_, for all 1 ≤ *i* ≤ *m*.

Given some *q*∈*U* that we wish to classify, we compare the similarity between its corresponding hypervector ϕ(*q*) and all class hypervectors ϕ(*C*_1_), ϕ(*C*_2_), …, ϕ(*C*_*m*_). The class whose corresponding class hypervector has the highest similarity to ϕ(*q*) is designated as the predicted class for *q*. Thus, the encoding scheme ϕ, dataset D, method of aggregating class hypervectors, and similarity measure δ(·, ·), together define a classification model based on HDC.

## 4 Estimating hyperdimensional robustness

### 4.1 Preliminary concepts and definitions

Before we discuss the robustness of such models, we first introduce the Random Fourier Feature (RFF) Rahimi and Recht (2007) encoding ϕ which is generally useful in the context of learning in HDC as it is an approximation of kernel methods. The RFF encoding is a map $\phi :{\mathbb{R}}^{n}\to C^{D}$, with ϕ(*x*) = *e*^*iMx*^, where each row *M*_*i*,:_ ~ *p* for some distribution *p*. As noted in the supplements, HDC is a general computation framework with various implementations. In this case, the resulting hypervectors mapped to by the RFF encoding operate under the Fourier Holographic Reduced Representation (FHRR) model of HDC, as FHRR base hypervectors are of the form *e*^*iθ*^, where θ is a column vector such that θ_*i*_ ~ *p*. Thus, using the language of HDC defined in the supplements, assuming each entry *M*_*ij*_ ~ *p*, we may also represent the mapping as $\phi \left(x\right)=H_{1}^{x_{1}}*H_{2}^{x_{2}}*...*H_{n}^{x_{n}}$, where $H_{i}=e^{iM_{:,i}}$ is an FHRR base hypervector and *M*_:, *i*_ is the *i*-th column of *M*. The similarity measure we use is the real component of the inner product defined on $C^{D}$ which we denote as 〈ϕ(*x*), ϕ(*y*)〉 = ℜ[ϕ(*x*)^*T*^ϕ(*y*)^*^], where ϕ(*y*)^*^ is the complex conjugate of ϕ(*y*). (Rahimi and Recht 2007) show, following from Bochner's theorem, that 〈ϕ(*x*), ϕ(*y*)〉/*D*≈*k*(*x*−*y*), where *k* is a shift-invariant kernel that is the Fourier transform of distribution *p*.

Next, before presenting our methods of estimating a model's robustness, we must precisely define what it means.

Definition 1 ((ϵ, *q*)-Robustness). A classifier *f* is (ϵ, *q*)-robust if *f*(*q*) = *f*(*q*+ω) for all ω such that ||ω|| ≤ ϵ. Alternatively, to denote the dependence of ϵ on *q*, we write ϵ_*q*_.

Note that our definition of (ϵ, *q*)-robustness is given with respect to some choice of norm ||·||.

The concept of (ϵ, *q*)-robustness is a notion of robustness that is *per data point*. It is easy to see that ϵ_*q*_ is the shortest distance from *q* to a decision boundary of *f* in the norm of choice. However, there are several practical considerations we have to make:

1. For even input spaces of moderately high dimensions, computing ϵ_*q*_ is in general infeasible.
2. In practice, we do not care about the robustness of a model for a single data point. Instead, we care about the robustness of a model for a dataset.

To address point 1, we will estimate ϵ_*q*_ using tractable methods. As we shall see in later subsections, there are various ways of estimating ϵ_*q*_ that have various levels of complexity and conservativeness. To address point 2, during the evaluation of our methods, rather than considering ϵ_*q*_ on a point-by-point basis, we will instead consider $E_{x\sim D}\left[\epsilon_{x}\right]$, where D is the dataset distribution.

In the following subsections, for simplicity, we will consider the binary classification case, although it is easy to extend our results to multi-class classification.

### 4.2 Linear approximation approach

Suppose we have two classes *C*_1_ and *C*_2_. We denote their corresponding class hypervectors as ϕ(*C*_1_) and ϕ(*C*_2_), respectively. Suppose we use the inner product as our similarity measure. Let *f* be the corresponding classifier; i.e., *f*(*x*) = 1 if 〈ϕ(*C*_1_), ϕ(*x*)〉≥〈ϕ(*C*_2_), ϕ(*x*)〉 and *f*(*x*) = 2 otherwise. We define the following function:


(1) $$\begin{array}{l} r\left(x\right)=\langle \phi \left(C_{1}\right)-\phi \left(C_{2}\right),\phi \left(x\right)\rangle . \end{array}$$

It is evident that *r*(*x*) = 0 corresponds to the decision boundary of *f*. Thus, a simple way of estimating the distance of some input *q* to the decision boundary is to take the linear approximation of *r* at *q* and compute its distance to zero. That is,


(2) $$\begin{array}{l} r\left(q\right)+\nabla r{\left(q\right)}^{T}x=0. \end{array}$$

Rearranging terms, we get


(3) $$\begin{array}{l} r\left(q\right)=-\nabla r{\left(q\right)}^{T}x. \end{array}$$

Taking absolute values and applying the Cauchy-Schwarz inequality, we get


(4) $$\begin{array}{l} |r\left(q\right)|=|\nabla r{\left(q\right)}^{T}x|\leq ||\nabla r\left(q\right)||||x||. \end{array}$$

Thus, we get the estimate


(5) $$\begin{array}{l} \epsilon_{q}\approx \frac{\left|r\left(q\right)\right|}{\left||\nabla r\left(q\right)|\right|}. \end{array}$$

There are various issues with this approach. First, there is no guarantee that the resulting ϵ_*q*_ computed this way satisfies the conditions for (ϵ, *q*)-robustness. Second, if *q* is close to some point in *C*_1_∪*C*_2_, this estimate of ϵ_*q*_ is quite likely to be a drastic overestimate as the gradient at those points tends to be close to zero. In this sense, it is a liberal estimate. So, this estimate is only useful for *q*'s close to the decision boundary, which is not very useful.

### 4.3 Lipschitz-based approach

For some applications, it is important to have a strong theoretical estimate for (ϵ, *q*)-robustness. The approaches explored in this subsection can achieve this in the ideal case. Central to these approaches is the concept of Lipschitz continuity.

Definition 2 (Lipschitz continuity). A function *f*:*X*→*Y* is Lipschitz continuous if there is some *L*>0 such that ||*f*(*x*)−*f*(*y*)||_*Y*_ ≤ *L*||*x*−*y*||_*X*_, where ||·||_*X*_ and ||·||_*Y*_ are distance measures for *X* and *Y*, respectively. The smallest such *L* is called the Lipschitz constant.

Thus, if a function is Lipschitz continuous, there is a bound on how fast it can change. If we are able to find some *L* for the function *r* defined in Equation 1, we can get an estimate for ϵ_*q*_ that satisfies (ϵ, *q*)-robustness. This is formalized by the following proposition.

Proposition 1. If *L* satisfies the Lipschtiz condition for *r*, then *f* is (ϵ, *q*)-robust for ϵ = |*r*(*q*)|/*L*.

*Proof*. Suppose *L* satisfies the Lipschtiz condition for *r*. Let *q* be some input query and ω some noise added to *q*, with ||ω|| ≤ ϵ. Then,


(6) $$\begin{array}{l} |r\left(q+\omega \right)-r\left(q\right)|\leq L||\omega ||\leq |r\left(q\right)|. \end{array}$$

Suppose *f* is not (ϵ, *q*)-robust. Then, there is some ω with ||ω||_2_ ≤ ϵ such that *r*(*q*+ω)≥0 and *r*(*q*) < 0 or *r*(*q*+ω) < 0 and *r*(*q*)≥0. It follows that


(7) $$\begin{array}{l} |r\left(q+\omega \right)-r\left(q\right)|>|r\left(q\right)|, \end{array}$$

which is a contradiction, thus proving our proposition.

Thus, with this result, our goal now is to find some *L* satisfying the Lipschitz condition for *r* to estimate ϵ_*q*_. Assuming *r* is differentiable, there is a basic result for finding *L* that follows from the Mean Value Theorem:

Proposition 2. If *r* is differentiable, then $L=\underset{x}{\sup}||\nabla r\left(x\right)||$ is its Lipschitz constant.

Taking $L=\underset{x}{\sup}||\nabla r\left(x\right)||$ is the best we can do, in the sense that it gives us the largest possible estimate for ϵ_*q*_ = |*r*(*q*)|/*L* that is (ϵ_*q*_, *q*)-robust using our Lipschitz-based approach. This gives us the estimate


(8) $$\begin{array}{l} \epsilon_{q}\approx \frac{\left|r\left(q\right)\right|}{\sup_{x}||\nabla r\left(x\right)||}. \end{array}$$

Unfortunately, in practice, solving for the global maximum of ||∇*r*(*x*)|| is intractable. The best we can do is to solve for local maxima. Thus, the estimate of ϵ_*q*_ we obtain in this way does not guarantee (ϵ_*q*_, *q*)-robustness, but it at least should not give drastic overestimates as in Equation 5. In this way, Equation 8 is a more conservative estimate.

### 4.4 Conservative Lipschitz-based approach

For this approach, we derive an expression for *L* that can be more easily computed via an easier optimization problem compared to the previous $L=\underset{x}{\sup}||\nabla r\left(x\right)||$. The result is given in the following proposition:

Proposition 3. *L* = α||ϕ(*C*_1_)−ϕ(*C*_2_)||_*H*_ satisfies the Lipschitz condition for *r*, where α satisfies the Lipschitz condition for the encoding ϕ. ||·||_*H*_ refers to the norm defined by the inner product 〈·, ·〉.

*Proof*. For any inputs *x, y*,


$$\begin{array}{l} \frac{\left|r\left(x\right)-r\left(y\right)\right|}{\left||x-y|\right|}=\frac{\left|\langle \phi \left(C_{1}\right)-\phi \left(C_{2}\right),\phi \left(x\right)-\phi \left(y\right)\rangle \right|}{\left||x-y|\right|}\\ \;\leq \frac{||\phi \left(C_{1}\right)-\phi \left(C_{2}\right)|{|}_{H}||\phi \left(x\right)-\phi \left(y\right)|{|}_{H}}{\left||x-y|\right|}\\ \;\leq \alpha ||\phi \left(C_{1}\right)-\phi \left(C_{2}\right)|{|}_{H} \end{array}$$

This gives us the estimate


(9) $$\begin{array}{l} \epsilon_{q}\approx \frac{\left|r\left(q\right)\right|}{\alpha ||\phi \left(C_{1}\right)-\phi \left(C_{2}\right)|{|}_{H}}. \end{array}$$

Now, we have delegated the problem to computing some α that satisfies the Lipschitz condition for ϕ. Note that Proposition 2 implies that *r* is scalar-valued, which is not the case for ϕ. Thus, we cannot use it to compute α. Instead, we will compute α on a case-by-case basis for each encoding ϕ.

We show how one can find α for any shift-invariant encoding where encoded hypervectors are of constant length; i.e., any encoding ϕ where 〈ϕ(*x*_1_), ϕ(*y*_1_)〉 = 〈ϕ(*x*_2_), ϕ(*y*_2_)〉 if *x*_1_−*y*_1_ = *x*_2_−*y*_2_ and ||ϕ(*x*)||_*H*_ = *K* for some *K*, for all *x*.

Proposition 4. Suppose ϕ is shift-invariant and ||ϕ(*x*)||_*H*_ = *K* for some *K*, for all *x*. Then, α satisfies the Lipschitz condition for ϕ, where


$$\begin{array}{l} \alpha =\underset{x}{\sup}\frac{\sqrt{2\left(K^{2}-\langle \phi \left(x\right),\phi \left(0\right)\rangle \right)}}{\left||x|\right|} \end{array}$$

*Proof*. We want to find some α such that


(10) $$\begin{array}{l} ||\phi \left(x\right)-\phi \left(y\right)|{|}_{H}\leq \alpha ||x-y||. \end{array}$$

Note that


$$\begin{array}{l} ||\phi \left(x\right)-\phi \left(y\right)|{|}_{H}=\sqrt{\langle \phi \left(x\right)-\phi \left(y\right),\phi \left(x\right)-\phi \left(y\right)\rangle }\\ \;=\sqrt{||\phi \left(x\right)|{|}_{H}^{2}+||\phi \left(y\right)|{|}_{H}^{2}-2\langle \phi \left(x\right),\phi \left(y\right)\rangle }\\ \;=\sqrt{2\left(K^{2}-\langle \phi \left(x\right),\phi \left(y\right)\rangle \right)}. \end{array}$$

By shift invariance, we have


$$\begin{array}{l} \frac{\sqrt{2\left(K^{2}-\langle \phi \left(x\right),\phi \left(0\right)\rangle \right)}}{\left||x|\right|}\leq \alpha . \end{array}$$

So, it is clear that


$$\begin{array}{l} \alpha =\underset{x}{\sup}\frac{\sqrt{2\left(K^{2}-\langle \phi \left(x\right),\phi \left(0\right)\rangle \right)}}{\left||x|\right|} \end{array}$$

satisfies the Lipschitz condition.

Compared to the optimization problem in the previous subsection given in Proposition 2, which depends on both the encoding and the dataset, the optimization problem here depends only on the encoding. For shift-invariant encodings where 〈ϕ(*x*), ϕ(0)〉 is approximately symmetric about the origin, it follows that the global maximum if it exists, should be close to the origin. This fact indicates that this optimization problem is an easier one.

While one generally cannot guarantee convergence to the global maximum, it is likely that an optimization scheme can get rather close. Thus, the resulting approximation of ϵ_*q*_ given in Equation 9, loosely speaking, is close to (ϵ_*q*_, *q*)-robust. Of course, while we get this stronger estimate compared to the approximation in Equation 8, this estimate is comparatively more conservative.

## 5 Learning a robust hyperdimensional encoding

We discuss how the results of the previous section can be used to learn a robust encoding. An encoding ϕ generally depends on some set of parameters *M* sampled from a distribution *p*. An example of this is the RFF encoding ϕ(*x*) = *e*^*iMx*^, where each row *M*_*j*_ ~ *p*.

Thus, we can characterize a family of encodings by parameterizing the encoding itself ϕ as well as the distribution *p*_θ_ from which random samples *M* are drawn. We denote such dependencies via the notation ϕ_η_(·;η, *M*). We also denote this dependence in ϵ_*q*_ by writing ϵ_*q*_(η, *M*). Let $\bar{\epsilon }\left(\eta ,M\right)$ be the average estimated robustness ϵ_*q*_(η, *M*) over the dataset, i.e., *𝔼*_*x* ~ *D*_[ϵ_*x*_(η, *M*)]. Thus, our goal is to find parameters η^*^, θ^*^ such that


(11) $$\begin{array}{l} \eta^{*},\theta^{*}=\arg\underset{\eta ,\theta }{\max}E_{M\sim p_{\theta }}\left[\bar{\epsilon }\left(\eta ,M\right)\right]. \end{array}$$

To compute the gradient $\nabla_{\eta }E_{M\sim p_{\theta }}\left[\bar{\epsilon }\left(\eta ,M\right)\right]$, we can simply do a Monte-Carlo estimate:


(12) $$\begin{array}{l} \nabla_{\eta }E_{M\sim p_{\theta }}\left[\bar{\epsilon }\left(\eta ,M\right)\right]=E_{M\sim p_{\theta }}\left[\nabla_{\eta }\bar{\epsilon }\left(\eta ,M\right)\right]. \end{array}$$

We make a simplifying assumption that *M* ~ *p*_θ_ is equivalent to *M* = *f*_θ_(*Z*), where *Z* ~ *N*(0, *I*). This gives us the gradient


(13) $$\begin{array}{l} \nabla_{\theta }E_{Z\sim N\left(0,I\right)}\left[\bar{\epsilon }\left(\eta ,f_{\theta }\left(Z\right)\right)\right]=E_{Z\sim N\left(0,I\right)}\left[\nabla_{\theta }\bar{\epsilon }\left(\eta ,f_{\theta }\left(Z\right)\right)\right]. \end{array}$$

## 6 Results

We apply our theoretical results above to both synthetic and real datasets. In the synthetic case, we compare the per point robustness ϵ_*q*_ to the actual distance to the decision boundary and show that our Lipschitz methods satisfy (ϵ_*q*_, *q*)-robustness. In the real case, we apply our methodology for kernel learning and demonstrate that the average robustness increases under our optimization scheme. We also test the effectiveness of average robustness $\bar{\epsilon }$ as a measure of robustness by plotting the degradation in accuracy of the HDC models with different levels of average robustness when noise of increasing magnitude is added to data points.

### 6.1 Results on synthetic dataset

We test our theoretical results for both one- and two-dimensional synthetic datasets. Each dataset consists of two classes, each of which is generated via sampling from a Gaussian distribution.

**Figure 2:**
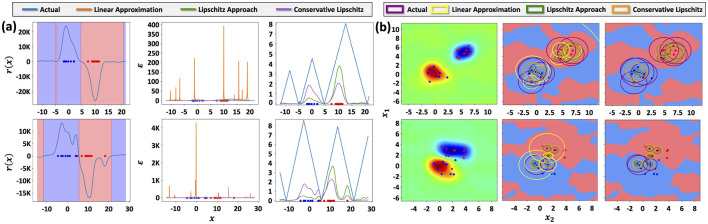
**(a)** Synthetic 1D dataset with two classes generated by sampling from Gaussian distributions. **(b)** Synthetic 2D dataset with two classes generated by sampling from Gaussian distributions. *Top row:* class samples are drawn from Gaussian distributions with low overlap. *Bottom row:* class samples are drawn from Gaussian distributions with higher overlap, leading to data that is not linearly separable.

Figure 2 visualizes (Figure 2a) 1D and (Figure 2b) 2D synthetic datasets alongside the decision function *r*(*x*), the decision regions, as well as the real and estimated robustness values; i.e., distance to the decision boundary. In the 1D case, this is visualized as the height of the plotted functions, and in the 2D case, as the radii of the circles. As can be seen, method 1 of computing ϵ_*q*_ does not give a good estimate of robustness as it tends drastically overestimate the distance to the decision boundary. Both Lipschitz-based methods give estimates that are generally less than the actual distance to the decision boundary, satisfying (ϵ, *q*)-robustness. In addition, we see that method 2 gives a better estimate in the sense that it is closer to the actual distance to the decision boundary, which corroborates with our theory above.

### 6.2 Results on visual data

**Figure 3:**
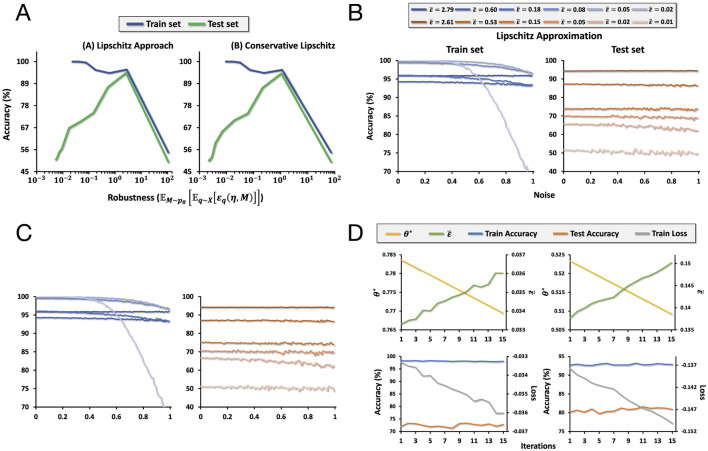
**(A)** The tradeoff between accuracy and robustness. Left: the tradeoff when accuracy is computed using method 2. Right: the tradeoff when the accuracy is computed using method 3. **(B)** A plot of the accuracy as increasing noise is added to the input space for models of different levels of average robustness computed using method 2. **(C)** A plot of the accuracy as increasing noise is added to the input space for models of different levels of average robustness computed using method 3. **(D)** Plots of loss, the length-scale parameter θ, average robustness, and accuracy over training iterations as we optimize for average robustness.

We use a binary classification version of MNIST where the dataset contains images of numbers 1 and 2. To encode each data point in the dataset, we use an RFF encoding with dimension 10,000, with random parameters *M* ~ *N*(0, σ*I*). Under our kernel learning framework, this can be expressed as *M* = *f*_σ_(*Z*) = σ*Z* where *Z* ~ *N*(0, *I*). Using the methodology described in the section above, we optimize σ to maximize the average robustness. Figure 3D shows that the average robustness increases over the number of iterations of the optimization process. We do this for both methods 2 and 3 of computing robustness described above.

At each iteration in the optimization process, we get a measure of average robustness based on methods 2 or 3. In addition, we compute the corresponding train and test accuracy of the model at that point. We visualize this in Figure 3A, which plots the tradeoff between train and test accuracy and robustness computed using methods 2 and 3. The train accuracy clearly falls as robustness increases. However, increasing robustness increases test accuracy up to a certain point, which then decreases as in the case of train accuracy. Thus, we may think of robustness as a kind of regularization.

For models of different levels of average robustness, we plot its degradation in train and test accuracy as we add increasing magnitudes of noise to the data in the input space. We visualize the results in Figures 3B, C. As average robustness increases, the degradation in train accuracy decreases more slowly as noise of increasing magnitude is added to the input space. In the case of test accuracy, while there is no clear trend as in the case of train accuracy, we do see that there is greater variance in accuracy across different magnitudes of noise for models with lower average robustness, while the quality is nearly constant for the model with the highest robustness.

## 7 Discussion

### 7.1 Extending beyond the RFF encoding

While we have illustrated our method of computing robustness estimates using HDC classifiers using the RFF encoding, our method extends to all HDC encoders that are Lipschitz-continuous. This result is highlighted in Proposition 3, which delegates the estimation of the robustness to estimating the Lipschitz constant of the HDC encoder of choice. It is important to note that this result requires the HDC encoder to be Lipschitz-continuous, which is a rather loose assumption, applying not only to differentiable encoders but also to non-differentiable ones. We outlined a method to estimate the Lipschitz constant for shift-invariant encoders (e.g., RFF) in Proposition 4 as a specific example, but it is possible to derive similar results for other HDC schemes, such as binary splatter codes (BSCs) (Kanerva, 1997), multiply add permute (MAP) (Gayler, 1998), matrix binding of additive terms (MBAT) (Gallant and Okaywe, 2015), and generalized holographic reduced representations (GHRRs) (Yeung et al., 2024). Investigating ways to estimate the Lipschitz constant for such encoders will be left for future work.

### 7.2 Quantized setting and neuro-vector-symbolic pipelines

As HDC is commonly considered within a quantized setting due to its plethora of hardware applications, we discuss a direction in which to extend our method to this particular setting. As noted above, our derived robustness estimate depends on the encoder's Lipschitz-continuity. In the quantized setting (e.g., using BSC or MAP schemes), core symbolic operations (e.g., XOR binding and fixed permutations) are Hamming isometries, so the dominant contribution to the Lipschitz constant comes from the encoder and any subsequent quantizer, which we can model as *e* = *q*°*f*, where *f* maps inputs to a continuous hypervector and *q* performs quantization. When *f* is *L*_*f*_-Lipschitz and *q* is non-expansive (deterministically or in expectation) under Hamming distance, one obtains a usable bound *L*_*e*_ ≤ *L*_*q*_*L*_*f*_, allowing our analysis to carry over naturally to this setting.

This decomposition aligns with established “translation” pipelines that move signals from neural or symbolic spaces into hypervector space. In particular, Mitrokhin et al. (2020) map learned embeddings to binary hypervectors and classify via binding/bundling, while Sutor et al. (2022) (HD-Glue) convert penultimate-layer signals from heterogeneous neural networks into hypervectors and aggregate them. Both follow a similar structural pattern of *e* = *q*°*f*: a continuous feature extractor (*f*) followed by a mapping to a discrete symbolic representation (*q*).

This approach also highlights a possible way of extending our method to translate to a neuro-vector-symbolic pipeline. While our theoretical results in this work are restricted to classifiers that operate entirely within the hypervector space (i.e., purely HDC-based), extending our results to guarantees to neuro-vector-symbolic pipelines that prepend a neural embedding requires accounting for the neural component's Lipschitz constant. Writing *f* = *h*°*g* with *g* a neural embedding and *h* a continuous HDC encoder gives us *L*_*e*_ ≤ *L*_*q*_*L*_*h*_*L*_*g*_ so finding the Lipschitz constant of the resulting encoder reduces to bounding *L*_*g*_ in addition to *L*_*h*_ and *L*_*q*_. Practical methods for controlling or estimating *L*_*g*_ are well studied (e.g., operator-norm bounds and spectral constraints at the layer level, orthogonal/Parseval parameterizations, and related Lipschitz-controlled architectures) and can be composed to produce conservative global bounds.

## 8 Conclusion

In summary, this study presents a first-of-its-kind theoretical framework for evaluating the robustness of hyperdimensional classifiers based on Lipschitz continuity, which can then be used as an optimization objective for learning more robust encodings. Our experimental results demonstrate the effectiveness of our approach. We believe our work can lead to the development of more robust and reliable hyperdimensional computing models and pave the way for further research in this area.

## Data Availability

The original contributions presented in the study are included in the article/supplementary material, further inquiries can be directed to the corresponding author.

## Footnotes

^1^Note that we are overloading our notation here, as *C*_*i*_∉*U*.
